# A Novel Thermostable and Processive Reverse Transcriptase from a Group II Intron of *Anoxybacillus flavithermus*

**DOI:** 10.3390/biom14010049

**Published:** 2023-12-29

**Authors:** Igor P. Oscorbin, Maxim L. Filipenko

**Affiliations:** Institute of Chemical Biology and Fundamental Medicine, Siberian Branch of the Russian Academy of Sciences (ICBFM SB RAS), 8 Lavrentiev Avenue, 630090 Novosibirsk, Russia; mlfilipenko@gmail.com

**Keywords:** reverse transcriptase, group II intron, *Anoxybacillus flavithermus*, thermal stability, processivity, reverse transcription, RT-LAMP

## Abstract

Reverse transcriptases (RTs) are a family of enzymes that synthesize DNA using an RNA template and are involved in retrovirus propagation and telomere lengthening. In vitro, RTs are widely applied in various methods, including RNA-seq, RT-PCR, and RT-LAMP. Thermostable RTs from bacterial group II introns are promising tools for biotechnology due to their higher thermostability, fidelity, and processivity compared to commonly used M-MuLV RT and its mutants. However, the diversity of group II intron-encoded RTs is still understudied. In this work, we biochemically characterized a novel RT from a thermophilic bacterium, *Anoxybacillus flavithermus*, which was isolated from a hot spring in New Zealand and has an optimal growth temperature of around 60 °C. The cloned RT, named Afl RT, retained approximately 40% of the specific activity after a 45 min incubation at 50 °C. The optimal pH was 8.5, the optimal temperature was between 45 and 50 °C, and Mn^2+^ ions were found to be an optimal cofactor. The processivity analysis with MS2 phage gRNA (3569 b) demonstrated that Afl RT elongated fully up to 36% of the template molecules. In reverse transcription and RT-qLAMP, the enzyme allowed up to 10 copies of MS2 phage genomic RNA to be detected per reaction. Thus, Afl RT holds great potential for a variety of practical applications that require the use of thermostable and processive RTs.

## 1. Introduction

Reverse transcriptases (RTs) are a specific family of DNA polymerases that synthesize DNA using RNA as a template. In eukaryotes, RTs are involved in the propagation of retroviruses and hepadnaviruses, maintenance of telomeres, and expansion of mobile genetic elements. In prokaryotes, several classes of RTs primarily serve to mediate various anti-phage immunity mechanisms. RTs are essential tools in various applications conducted in vitro, such as research, biotechnology, and molecular diagnostics.

Among the numerous known RTs, the enzyme most commonly utilized is Moloney murine leukemia virus reverse transcriptase (M-MuLV RT). This enzyme is relatively well-studied. Its mutated variants exhibit increased thermal stability, tolerance to inhibitors, and processivity and are commercially available. Other popular commercial RTs are avian leukemia virus RT (AVL RT) and HIV-1 RT, both derived from retroviruses. However, retroviruses are not the only source of reverse transcriptases: RTs have also been found in prokaryotes. Over the recent few decades, a number of bacterial RTs have been identified, including enzymes from group II introns (GII introns) [1], retrons [2], diversity-generating retroelements (DGR) [3], abortive bacteriophage infection (Abi) [4,5], and CRISPR-Cas systems [6]. Except for GII introns, these genetic elements are involved in bacterial defense against phages, with the mechanism of action involving reverse transcription [6,7,8,9]. The exact functions of several other smaller classes of RTs still have to be identified. Approximately 40% of complete and draft genomes contain at least one gene encoding putative RT. Thus, reverse transcriptases in prokaryotes are highly diverse, and their roles in cells are still to be comprehensively elucidated.

Group II introns are frequent retroelements residing in bacterial and eukaryotic genomes. Their propagation mechanism involves combining autocatalytic self-splicing RNA with an intron-encoded protein (IEP) with enzymatic activities. Group II introns are thought to be ancestral to spliceosomes and eukaryotic non-LTR retrotransposons [10,11]. Thus, these retroelements are crucial for studying the evolution of eukaryotic genomes and splicing mechanisms. However, group II introns may also be involved in other cellular processes, namely, double-strand break repair. The biochemical features of RT from *Pseudomonas aeruginosa* are similar to those of the translesion DNA polymerase θ, with RT involved in DNA repair in both host and *E. coli* cells [12]. In turn, DNA polymerase θ also possesses reverse transcriptase activity and works faster using RNA as a template [13]. Taken together, these factors suggest that group II introns and cognate non-LTR retrotransposons may play a role in the process of DNA repair.

In group II introns, RTs participate in the propagation of mobile genetic elements by replicating their long coding sequences. GII-RTs demonstrated superior fidelity and processivity compared to retroviral RTs, resulting in more uniform coverage in RNA-seq than when using classical retroviral RTs [14,15]. Bacterial RTs also possess strong template-switching ability, which is beneficial for adaptor attachment in RNA-seq and makes it possible to dispense with RNA ligation or tailing steps. RTs from thermophilic bacteria are thermostable enzymes that allow cDNA synthesis to be performed at temperatures around and above 50–60 °C. Higher reaction temperatures destabilize RNA secondary structures, which may lead to RT stalling and, consequently, to inefficient synthesis with short products. For these reasons, thermostable RTs can be used for single-tube analysis performed at a constant temperature. Thus, a simple and cost-effective method of point-of-care testing becomes possible. Considering all the above advantages, RTs from bacterial group II introns, especially from thermophilic bacteria, are promising tools for practical applications.

To date, several RTs or intron encoded proteins have been reported from bacterial group II introns of *Thermosynechococcus elongates* [14], *Geobacillus stearothermophilus* [14,16], *Eubacterium rectale* [15], *Oceanobacillus iheyensis* [17], *Lactococcus lactis* [18], *Sinorhizobium meliloti* [19], *Thermoanerobacter italicus* [20], *Bacillus halodurans* [21], and *Pseudomonas aeruginosa* [12]. RTs from *L. lactis*, *T. elongates*, *G. stearothermophilus*, and *E. rectale* were biochemically characterized and tested for practical applications. In other cases, reverse transcriptase activity was detected, but information about optimal reaction conditions has not been published yet. Thus, the wide diversity of bacterial RTs still needs to be discovered. In this regard, thermophilic bacteria could prove to be a good source of new, effective RTs.

In the present study, we cloned and characterized a novel reverse transcriptase from a group II intron of thermophilic bacteria named Afl RT, *Anoxybacillus flavithermus*. The enzyme demonstrated optimal temperature around 45–50 °C and high processivity. Afl RT was also suitable for RT-qPCR and RT-qLAMP, providing a sensitivity of up to 10 MS2 phage genomic RNA molecules per reaction.

## 2. Materials and Methods

### 2.1. Search and Selection of Afl RT Coding Sequence

The MyRT tool from Sharifi and Ye was used to select reverse transcriptase genes [22]. This tool allows the selection of putative genes of reverse transcriptases in all complete and bacterial genomes. The search was conducted in RTs of a GII class from draft and complete genomes based on the presence of the RVT_1 domain (Pfam ID: PF00078), which is common for all known RTs. All the selected GII candidate RTs were curated, and only those from thermophilic hosts were further considered. The last step was to select only RTs without additional domains, except for GIIM (maturase), for cloning.

### 2.2. Expression and Purification of Afl RT

The coding sequence of Afl RT was synthesized and cloned into a pET28b vector by Shanghai RealGene Bio-tech, Inc. (Shanghai, China), resulting in a plasmid pAflRT.

A starter culture of *E. coli* BL21 (DE3) pLysS (Promega, Madison, WI, USA) strain harboring the plasmid pAflRT was grown to OD_600_ = 0.3 in LB medium with 25 μg/mL kanamycin at 37 °C. In a LiFlus GX fermenter (Biotron Inc., Bucheon, Republic of Korea), 4 L of LB with 25 μg/mL kanamycin were inoculated with 40 mL of the starter culture, and the cells were grown to OD_600_ = 0.6 at 37 °C. The expression of Afl RT was induced by adding IPTG up to 1 mM concentration. After induction for 4 h at 37 °C, the cells were harvested by centrifugation at 4000× *g* and stored at −70 °C.

For protein purification, the cell pellet was resuspended in a lysis buffer (50 mM Tris-HCl pH 9.0, 100 mM NaCl, 1 mM PMSF, 2 M urea, 1 mg/mL lysozyme) and incubated for 30 min on ice followed by sonication. After lysis, the soluble fraction was separated by two consequent centrifugation steps at 20,000× *g* for 30 min. Soluble proteins were precipitated ON by 60% (NH_4_)_2_SO_4_, followed by centrifugation at 20,000× *g* for 30 min. The resulting pellet was suspended in lysis buffer and loaded onto a 5 mL IMAC column (Bio-Rad, Hercules, CA, USA) pre-equilibrated with buffer A (50 mM Tris-HCl pH 8.0, 0.3 M NaCl), followed by washing the column with 25 mL of buffer A with 1 M NaCl. Bound proteins were eluted using 10 column volumes and a 0–100% linear gradient of buffer B (buffer A with 0.5 M imidazole). After affinity chromatography, the fractions with Afl RT were pooled and loaded onto a 2 mL Macro-Prep DEAE Resin (Bio-Rad, Hercules, CA, USA) pre-equilibrated with buffer C (50 mM Tris-HCl, 0.1 mM EDTA, pH 8.0). The column was washed with 10 mL of buffer C, and bound proteins were eluted by 10 column volumes and a 0–100% linear gradient of buffer D (50 mM Tris-HCl, 1 M NaCl, 0.1 mM EDTA, pH 8.0). The fractions with Afl RT were pooled, dialyzed against a storage buffer (20 mM Tris-HCl, 200 mM KCl, 0.1 mM EDTA, 50% glycerol, pH 8.5), and stored at −20 °C. All the fractions from each step were analyzed using SDS-PAGE. The purity of the isolated Afl RT was not less than 95%. The concentration of purified Afl RT was measured using a standard Bradford assay.

### 2.3. Reverse Transcriptase Activity Assay

The specific activity of Afl RT was assessed by using radiolabelled nucleotide incorporation. The reaction mix (50 µL) contained 0.4 mM poly(rA)/oligo(dT)25 (concentration defined by oligo(dT)42), 0.5 mM α-[32P]-dTTP (4 Bq/pmol), 50 mM Tris-HCI (pH 8.5), 6 mM MgCl_2_, 150 mM (NH_4_)_2_SO_4_, 5 mM DTT. The reactions were initiated by adding the enzyme on ice, with immediate transfer of the samples to a preheated thermal cycler for incubation at 45 °C for 10 min, followed by inactivation by heating at 90 °C for 1 min. The reaction products were collected on DE81 paper (Sigma-Aldrich, St. Louis, MO, USA), washed twice with 0.5 M Na_2_HPO_4_, and counted in a Pharos PX (Bio-Rad, Hercules, CA, USA). One unit of polymerase activity was defined as the amount of enzyme that incorporated 1 nmol of dTTP into acid-insoluble material in 10 min at 50 °C.

### 2.4. Thermal Stability, Optimal Temperature, and Ion Concentration

The thermal stability of Afl RT was studied by heating the enzyme and then assaying the reverse transcriptase activity. The aliquots of the polymerase activity reaction buffer (described above) containing an identical amount of 0.2 U of Afl RT were incubated at temperatures from 40 °C to 80 °C, increasing by 10 °C per step for 15–90 min. The reactions were chilled on ice, and the reverse transcriptase activity was measured as mentioned above.

The temperature optimum of Afl RT was defined by measuring the reverse transcriptase activity at temperatures from 25 °C to 70 °C, increasing by 5 °C per step, with other conditions identical to those described above for the reverse transcriptase activity assay. The reactions were initiated by adding the aliquots of enzymes to mixes containing all other components, including the primed template. The mixes were preheated for 5 min before the addition of enzymes.

The optimal ion concentrations were examined as in the reverse transcriptase activity assay using 25–400 mM NH_4_Cl, KCl, 50–400 mM NaCl, Na_2_SO_4_, (NH_4_)_2_SO_4_, or 1–10 mM BaCl_2_, CaCl_2_, FeSO_4_, MgCl_2_, MnCl_2_, ZnSO_4_, CoCl_2_, CuCl_2_, NiCl_2_.

### 2.5. cDNA Synthesis

The template RNA for cDNA synthesis was isolated from MS2 phage as described in the Supplementary Materials. The 10 µL reaction probes containing MS2 RNA and 25 µM random N7 were heated for 3 min at 65 °C, followed by cooling on ice at 5 min. The amount of MS2 RNA is specified for each experiment in the Results section. After priming, 10 µL of 2× reaction buffer (50 mM Tris-HCI (pH 8.5), 6 mM MgCl_2_, 150 mM (NH_4_)_2_SO_4_, 5 mM DTT) with 200 U of Afl RT were added to the cooled template-primer mixtures, and the probes were immediately transferred to a preheated thermal cycler for 1 h incubation at 50 °C, followed by heating at 90 °C for 3 min. The reaction products were analyzed using quantitative real-time PCR.

### 2.6. Quantitative PCR

Real-time RT PCR was performed in a CFX 96 thermocycler (Bio-Rad, Hercules, CA, USA) in a total reaction volume of 20 µL, containing a 1× PCR buffer (65 mM Tris-HCl pH 8.9, 24 mM (NH_4_)_2_SO_4_, 0.05% Tween 20, 2.5 mM MgCl_2_), 0.3 µM primers MS2-5-F/R and 0.1 µM probe MS2-5-P (Table 1), 1 unit of Taq-polymerase (Biosan, Novosibirsk, Russia) and a cDNA template as indicated in the Results section. The amplification was performed using the following program: denaturation at 95 °C for 3 min and 50 cycles with denaturation at 95 °C for 10 s, followed by annealing and elongation at 60 °C for 40 s with the registration of fluorescent signals in a FAM channel. Each experiment was conducted in three independent replicates, each run including a no-template control.

### 2.7. Reverse-Transcription Quantitative Loop-Mediated Isothermal Amplification (RT-qLAMP)

The reaction mixture for LAMP (20 µL) contained 1× reaction buffer for Bst-polymerase (20 mM Tris-HCl pH 8.8, 10 mM (NH_4_)_2_SO_4_, 50 mM KCl, 0.1% Tween-20, 8 mM MgSO_4_), 1.25 mM each dNTP, 0.4 µM each external primer (F3/B3), 0.8 µM loop primers (LF/BF), 1.6 µM internal primers (FIP/BIP) (Table 1), MS2 RNA as a template, 8 units of Gss-polymerase from *Geobacillus* sp. 777 [23], 200 U of Afl RT, and 1 µM intercalating dye SYTO-82. The amount of MS2 RNA is specified for each experiment in the Results section. RT-LAMP was performed in the CFX96 thermocycler (Bio-Rad, Hercules, CA, USA) using a two-step program with a 15 min initial reverse transcription, followed by real-time LAMP. The exact temperature of the reverse transcription step is specified below in the Results section for each experiment. The program included the following steps: 90 cycles of primer annealing and elongation, each at 62 °C for 30 s, with the registration of fluorescence signal in a HEX channel and post-amplification melting of amplification products in the range of 70–95 °C. Each experiment was conducted in three independent replicates, each run including a no-template control and a no-reverse transcriptase control. Tt values (time-to-threshold, time interval before the intersection between an amplification curve and a threshold line) were calculated after each run and were used to assess RT-qLAMP efficacy.

### 2.8. Processivity Assay

Reaction mixes in a 20 μL volume containing 5 ng MS2 RNA and 1 µM MS2-5-F2 primer (Table 1) were heated for 3 min at 65 °C, followed by cooling on ice at 5 min. After priming, 10 µL of 2× reaction buffer (50 mM Tris-HCI (pH 8.5), 6 mM MgCl_2_, 150 mM (NH_4_)_2_SO_4_, 5 mM DTT) with 200 U of Afl RT were added to cooled template-primer mixtures. Then, the probes were immediately transferred to a preheated thermal cycler for 1 h incubation at 50 °C, followed by heating at 90 °C for 3 min. The reaction products were analyzed using droplet digital PCR.

### 2.9. Droplet Digital PCR

The ddPCR was performed using the QX100 system (Bio-Rad, Hercules, CA, USA) according to the manufacturer’s recommendations. A 20 µL ddPCR reaction mixture contained 1 × ddPCR master mix (Bio-Rad, Hercules, CA, USA), 0.9 µM primers, 0.25 µM probe (Table 1) and 2 mL of tested cDNA. The entire reaction mixture and 70 µL of droplet generation oil (Bio-Rad, Hercules, CA, USA) were loaded into a disposable plastic cartridge (Bio-Rad, Hercules, CA, USA) and placed in a droplet generator. After processing, the droplets obtained from each sample were transferred to a 96-well PCR plate. The amplification was carried out using T100TM Thermal Cycler (Bio-Rad, Hercules, CA, USA) according to the program: DNA polymerase activation at 95 °C for 10 min followed by 45 cycles of PCR amplification (94 °C for 30 s and 58 °C for 60 s), and 98 °C for 10 min, 2 °C/s^−1^ ramp rate at all steps. After PCR, the droplets were counted using a QX100 Droplet Reader. The data obtained were analyzed using QuantaSoft Analysis Pro^TM^ 1.0.596 software (Bio-Rad, Hercules, CA, USA).

## 3. Results

### 3.1. Search for the Afl RT Gene and Purification of Afl RT

To search for a new RT from bacterial group II introns, we used an online tool reported in the work of Sharifi and Ye [22]. The tool allows one not only to predict novel RTs but also to search for putative reverse transcriptases among draft and complete genomes. We searched for novel GII RTs from thermophilic bacteria using the following criteria: the presence of the common for RTs RVT_1 domain (Pfam ID: PF00078), a thermophilic host, and the absence of any additional domains in an intron-encoded protein, with the exception of GIIM maturase. Thus, we attempted to select putative thermostable RTs without any additional unpredictable enzymatic activities that are often found among bacterial reverse transcriptases. Following these criteria, among 6776 GII RTs from complete and 6209 GII RTs from draft genomes, we selected a single enzyme from group II intron, belonging to a structural class IIC, of a thermophilic bacterium *Anoxybacillus flavithermus*. The host was found in a hot spring in New Zealand with an optimal temperature for growth around 60 °C [24]. Previously, several thermostable enzymes were isolated from *A. flavithermus*, including lipase [25], α-amylase [26], cyclomaltodextrinase [27], xylanases [28], with a temperature optimum in the range of 50–65 °C. Therefore, it was legitimate to assume the same thermostability of an RT from the same bacterium.

**Figure 1:**
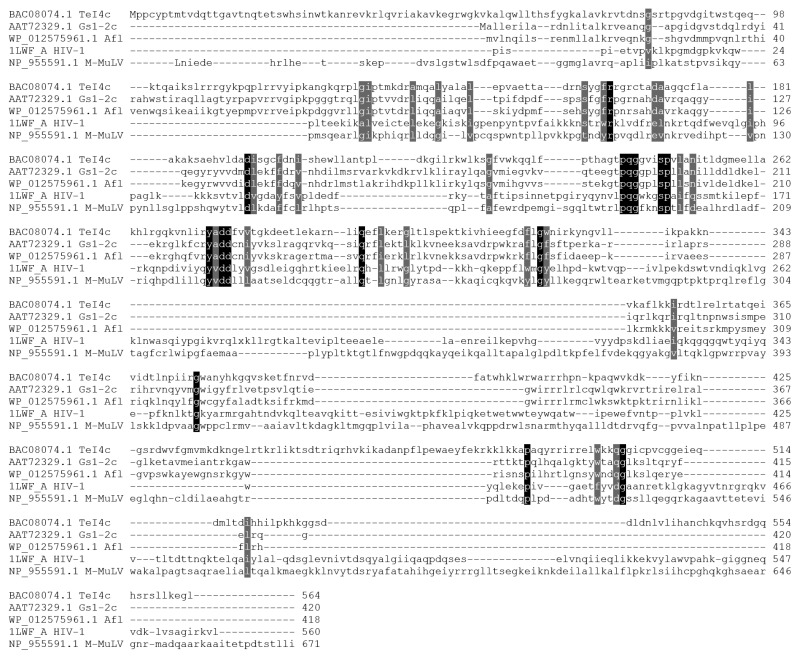
Alignment of several retroviral and bacterial GII RTs. Amino acid sequences were aligned using ClustalW and visualized in Sequence Manipulation Suite. The colored background indicates conservative amino acid residues.

The amino acid sequence of Afl RT was aligned with the commonly used retroviral M-MuLV RT and the previously characterized TeI4c RT from *Thermosynechococcus elongates* [14] and GsI-IIC RT from *Geobacillus stearothermophilus* [14,16] (Figure 1).

As shown previously, retroviral and bacterial GII RTs share only a small sequence similarity, indicating the evolutionary distance between these enzymes. Only a few conservative positions can be found in the alignment, such as the F/YxDD motif common for all reverse transcriptases, which binds magnesium cations necessary for catalysis. GII RTs lack the binding and the RNAseH domain involved in the processing of retroviral genomic RNA. Additionally, GII RTs are generally highly positively charged proteins with a net charge of 35–45 at pH 7.0 and pI values around 10.0–10.7, which can hinder purification of recombinant enzymes (Table 2).

**Figure 2:**
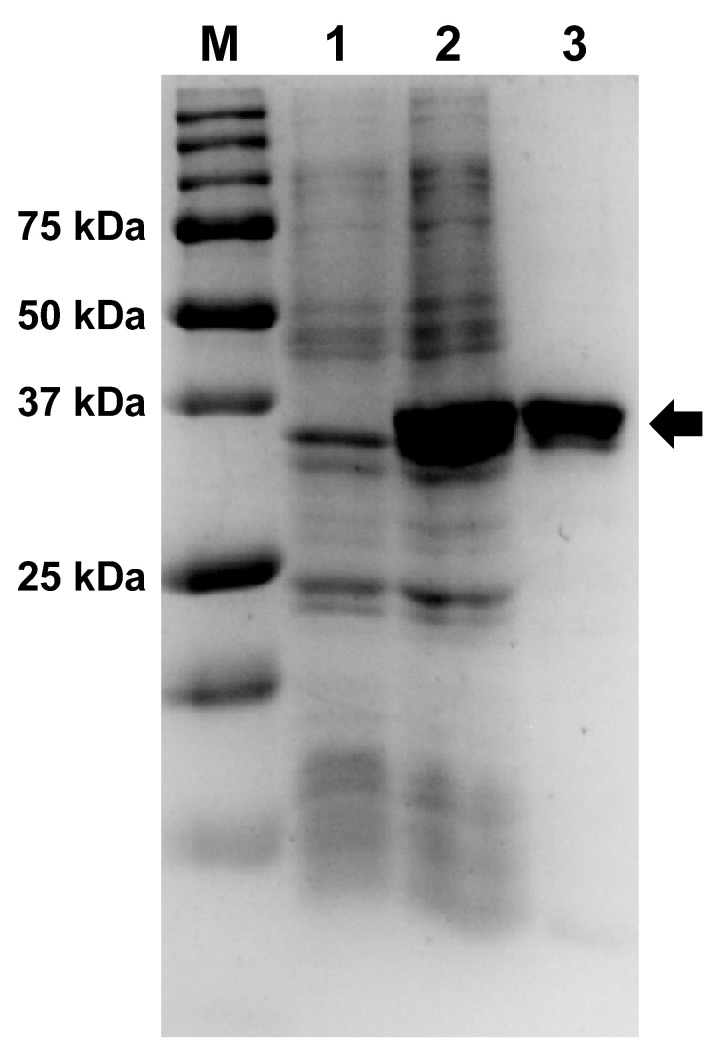
Expression and purification of Afl RT. The enzyme was expressed in *E. coli* strain BL-21 (DE3) pLysS and purified using affinity and ion-exchange chromatography. Lane assignment: M—Precision Plus Protein standards (Bio-Rad, Hercules, CA, USA), 1—*E. coli* lysate before expression of Afl RT, 2—crude lysate after expression, 3—purified Afl RT. Afl RT is marked with an arrow. Original images can be found in Figure S1.

Afl RT was expressed in *E. coli* BL21 (DE3) pLysS cells and purified using two chromatography steps on affinity and ion-exchange resins. The electrophoretic purity of the isolated enzyme was not less than 95% (Figure 2, Supplementary Materials, Figure S1). 

Surprisingly, Afl RT was relatively easily solubilized by adding 2 M urea in a buffer for *E. coli* cell lysis, whereas other purification conditions were not adjusted for highly charged proteins. No solubilization tags, such as MBP, were necessary to obtain active Afl RT. 

### 3.2. Biochemical Properties of Afl RT

#### 3.2.1. Optimal Temperature and Thermostability

After purification, we studied the biochemical properties of Afl RT using fluorescently labeled oligo(dT)_40_ primer and poly(r)A template. The molar activity of Afl RT was 1.58 × 10^5^, which is close to the molar activities of AVL RT and M-MuLV RT, reverse transcriptases from retroviruses [29]. 

**Figure 3:**
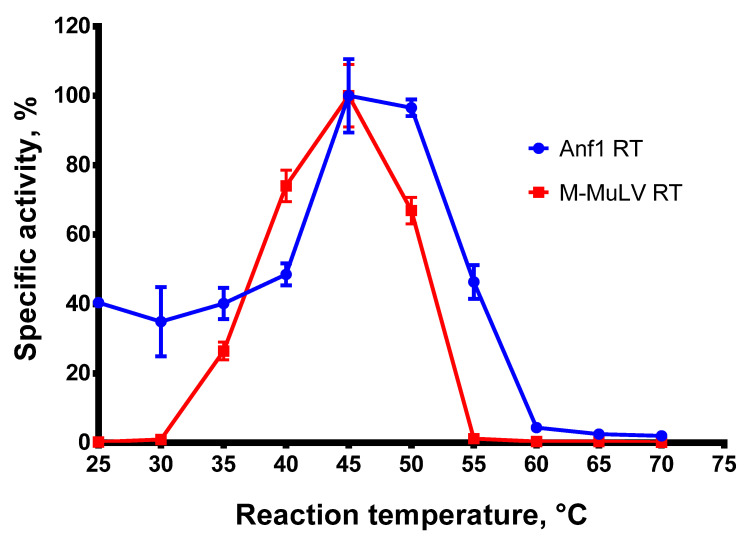
Optimal temperature assay. The optimal temperature for RTs was determined in RT activity assay using radioactively labeled oligo(dT)_40_ primer in poly(rA)/oligo(dT)_40_ substrate. Each experiment was triplicated.

A high reaction temperature is beneficial for reverse transcription, melting complex RNA structures, and increasing the specificity of RTs against mismatched primers. To examine the optimal reaction temperature for Afl RT, we carried out reverse transcription at 25–70 °C with a step of 5 °C and quantified the reaction products. The results of the optimal temperature assay are presented in Figure 3.

Afl RT demonstrated the highest reaction speed at 45–50 °C. This observation contradicts the optimal growth temperature of the host bacteria, *Anoxybacillus flavithermus*, which is around 60 °C. A possible reason for the discrepancy could be the stabilization of the reverse transcriptases by the substrates previously reported for M-MuLV RT [30]. A similar effect may occur for GII-RTs, which are known for their tight binding to the respective intron RNA. In further experiments, reverse transcription was performed at 50 °C.

**Figure 4:**
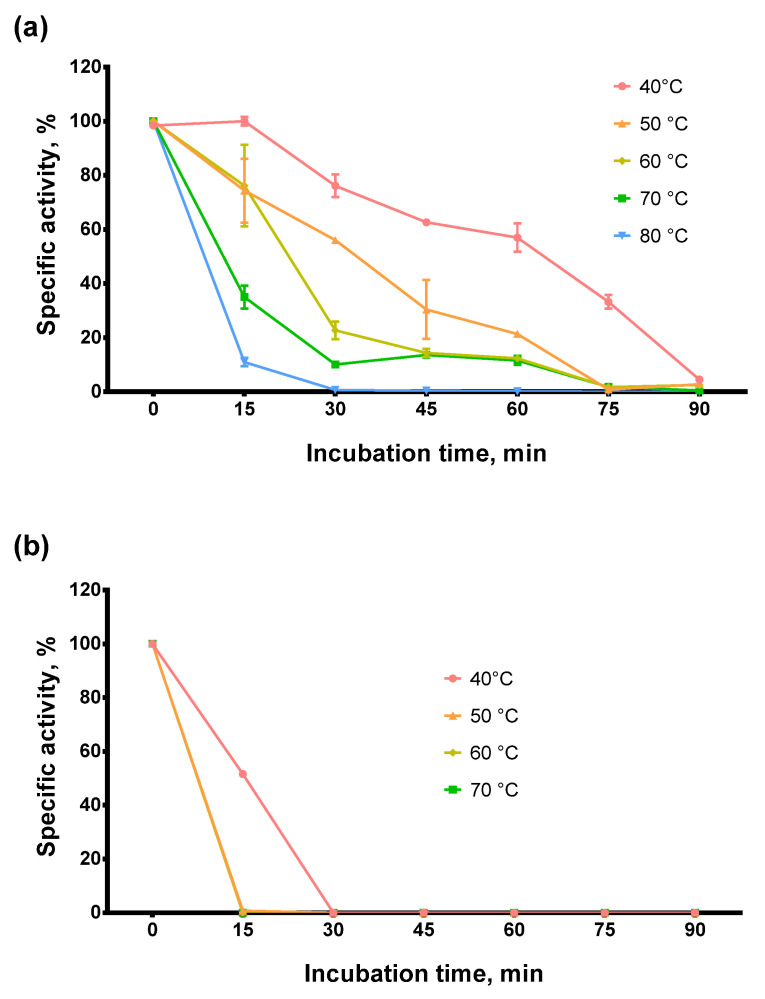
Thermostability assay. (**a**)—Afl RT, (**b**)—M-MulV RT. Enzymes were incubated at 40–80 °C for up to 90 min following RT activity assay using radioactively labeled oligo(dT)_40_ primer in poly(rA)/oligo(dT)_40_ substrate. Each experiment was triplicated.

Next, we tested the thermostability of Afl RT. Thermal stability is closely related to optimal reaction temperature and defines the ability of an enzyme to retain a specific activity after exposure to elevated temperatures. For examining the thermostability of Afl RT, the enzyme was incubated at 40–80 °C for up to 90 min following RT activity analysis. The results of the assay are presented in Figure 4.

The enzyme retained 60% of RT activity after incubation at 50 °C for 30 min or 60 min at 40 °C and 40% after 45 min at 50 °C. At higher temperatures, Afl RT lost the activity much faster: after 30 min at 70 °C, only 10% of activity was detected, and after 30 min at 80 °C, the reverse transcriptase was completely inactivated. Thus, Afl RT was relatively thermostable compared to M-MuLV RT. Note that we used Tris as a buffer reagent when evaluating optimal and thermal stability. Tris buffers decrease pH with increasing temperature. Although this temperature-dependent pH shift could have affected our results, we still chose to perform experiments with Tris. We were motivated by the widespread use of Tris in biotechnology. Thus, the thermostability and the optimal temperature in Tris may prove more helpful for specialists working in the field.

#### 3.2.2. Optimal Reaction Buffer

**Figure 5:**
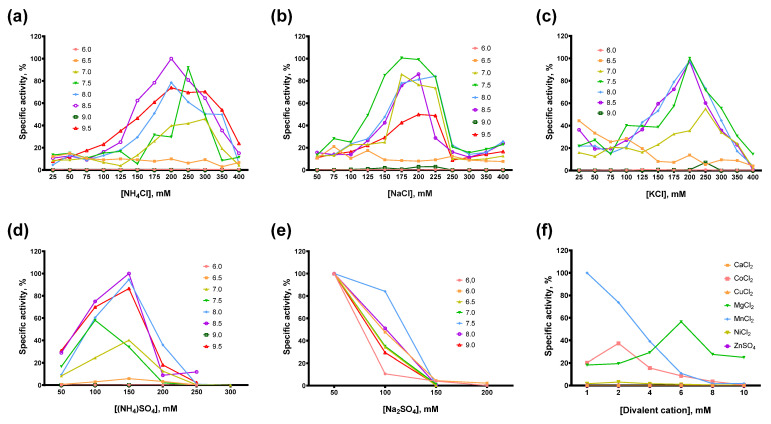
Optimal reaction buffer for Afl RT. Salts concentration and pH were varied in RT activity assay with radioactively labeled oligo(dT)_40_ primer in poly(rA)/oligo(dT)_40_ substrate: (**a**) NH_4_Cl, (**b**) NaCl, (**c**) KCl, (**d**) (NH_4_)_2_SO_4_, (**e**) Na_2_SO_4_, (**f**) divalent cations Ba^2+^, Ca^2+^, Co^2+^, Cu^2+^, Fe^2+^, Mg^2+^, Mn^2+^, Ni^2+^, Zn^2+^.

The composition of the reaction buffer, including pH, type and concentration of buffer reagent, salts, detergents, and other additives such as DTT, defines the efficacy of enzymatic catalysis. Ionic strength and pH affect the conformation and interaction of the enzyme and its substrate, resulting in favorable or unfavorable conditions for chemical transformations in the active site of the enzyme. Other critical factors are cofactors, such as divalent cations in the case of reverse transcriptases. To develop an optimal reaction buffer for Afl RT, we titrated several salts at various pH in reverse transcription after selecting the type and concentration of the optimal divalent cation. The concentration of the buffer reagent was the same in all the experiments: 50 mM Tris-HCl for pH 7.0–9.5 and 25 mM MES for pH 6.0–6.5. For divalent cations, the concentration of dNTP was fixed at 0.5 mM. The results of the titration experiments are presented in Figure 5.

**Figure 6:**
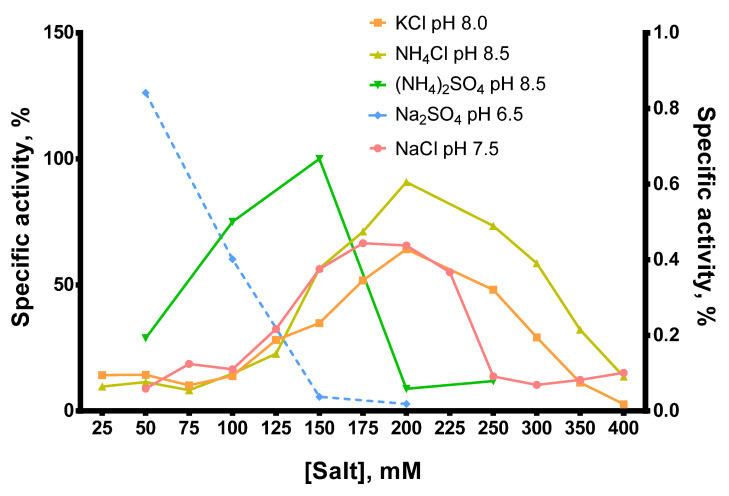
Comparison of optimal salts and pH for Afl RT. RT activity assay with radioactively labeled oligo(dT)_40_ primer in poly(rA)/oligo(dT)_40_ substrate. The dashed line (Na_2_SO_4_, pH 6.5) belongs to the right Y-axis.

In most cases, the optimal salt concentration was independent of the pH of the reaction buffer. Only for NH_4_Cl, KCl, and pH 7.0, the optimal salt concentration was 50–100 mM higher than for other pH values. The optimal concentration of sulfates was lower than that of chlorides: 200–250 mM NH_4_Cl and 150 mM (NH_4_)_2_SO_4_, 200–250 mM NaCl and 50 mM Na_2_SO_4_, respectively. K_2_SO_4_ was not titrated because of the high ionic strength and relatively low solubility of this sulfate. When different salts were compared, the highest activity of Afl RT was observed with 150 mM (NH_4_)_2_SO_4_ and pH 8.5 which was used in all subsequent experiments. It should be noted that the activity of Afl RT with Na_2_SO_4_ was low, approximately 1% of that with (NH_4_)_2_SO_4_ (Figure 6).

Among the divalent cations, Afl RT was completely inactive with CuCl_2_, ZnSO_4_. With CaCl_2_, only a residual activity of less than 1% of the specific activity with MgCl_2_ was observed. The inhibitory effect of NiCl_2_ was less striking, with Afl RT retaining around 5% of activity compared to MgCl_2_. Despite the low activity with NiCl_2_ and total inhibition by FeSO_4_, Afl RT was surprisingly active with CoCl_2_: the activity with 2 mM CoCl_2_ reached 66% of the activity with 6 mM MgCl_2_. The most favorable cofactor for Afl RT was MnCl_2_ instead of MgCl_2_: the enzyme was 1.75-fold active with MnCl_2_. However, in subsequent experiments, we used 6 mM MgCl_2_ due to the higher error rate of the synthesis with Mn^2+^ cations, which was already shown for other RTs [31,32,33]. Additionally, Afl RT synthesized a greater amount of cDNA in the presence of 5 mM DTT.

### 3.3. Afl RT in Practical Applications

From a practical point of view, assessing RT in a variety of applications is of importance. Novel enzymes may be more efficient than popular commercial reverse transcriptases and may become valuable tools for modern biotechnology. Here, we chose reverse transcription, RT-PCR, and RT-LAMP as the most common approaches using RTs. The genomic MS2 strand RNA served as a template and was titrated in the range of 1 × 10^2^–1 × 10^8^ copies per reaction.

#### 3.3.1. Afl RT in Reverse Transcription

**Figure 7:**
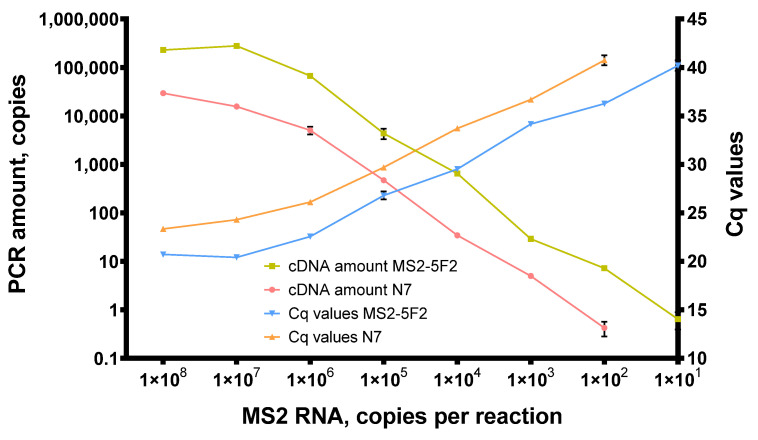
Titration of MS2 genomic RNA in reverse transcription with Afl RT. Reverse transcription was conducted with 1 × 10^2^–1 × 10^8^ of MS2 genomic RNA as a template at 50 °C for 40 min with random hexamers and 200 U of Afl RT per reaction. Reaction products were quantified by qPCR. cDNA amount and Cq values were plotted against the template concentration. Each experiment was triplicated.

In reverse transcription, 200 U of Afl RT was used per reaction with random N7 primers, and the reaction mixes were incubated at 50 °C for 1 h. After reverse transcription, the amount of cDNA was quantified by qPCR and is plotted in Figure 7.

With N7 primers, Afl RT successfully synthesized cDNA when down to 1 × 10^2^ copies of MS2 genomic RNA were added in reverse transcription mixes. With a specific MS2-5F2 primer, reverse transcription proved approximately 10 times more effective. Thus, the same amount of the template yielded more cDNA, with the least detectable template concentration being 10^1^ copies of MS2 genomic RNA per reaction. This higher efficacy of reverse transcription with the specific primer could have been due to its higher melting temperature. N7 primers are much shorter and most of them cannot anneal at 50 °C.

#### 3.3.2. Afl RT in RT-qLAMP

**Figure 8:**
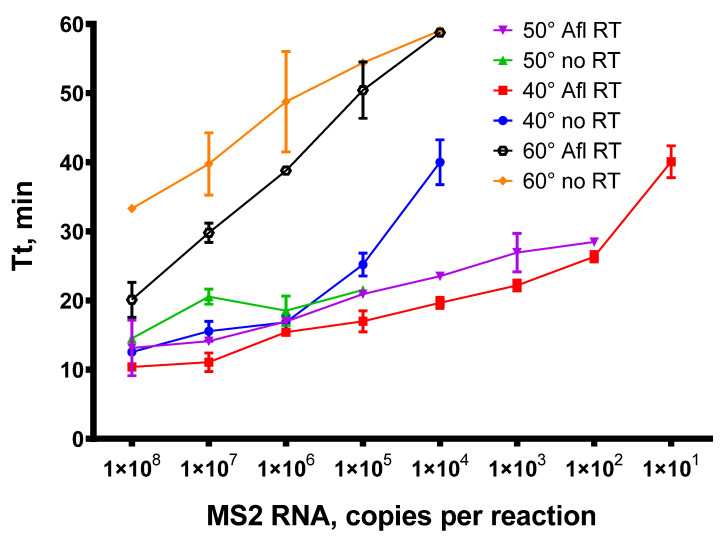
Titration of MS2 genomic RNA in RT-qLAMP with Afl RT. RT-qLAMP was conducted with 1 × 10^2^–1 × 10^8^ of MS2 genomic RNA as a template and 200 U of Afl RT per reaction. The temperature of a reverse transcription step was 40, 50, or 60 °C for 15 min. Time-to-threshold values were plotted against a template concentration. Each experiment was triplicated.

In the RT-qLAMP assay, a total of 200 U of Afl RT was used per reaction. The reverse transcription step was conducted at 40, 50, or 60 °C. The sequences for LAMP primers MS2-F3/B3/LB/LF/FIP/BIP are given in Table 1. With each temperature and template concentration, a control reaction was performed without Afl RT to test the reverse transcriptase activity of Gss-polymerase. After the completion of RT-qLAMP, LAMP products were examined by analyzing the melting curves. The time-to-threshold (Tt) values obtained are presented in Figure 8.

Similar to reverse transcription, the addition of Afl RT in RT-qLAMP enabled the detection of up to 10^1^ copies of MS2 genomic RNA when the temperature of reverse transcription was 40 °C and 10^2^ copies per reaction when the temperature was 50 °C. In the absence of specific reverse transcriptase, RT-qLAMP exhibited significantly reduced sensitivity, with a detection limit of 10^5^ at 50 °C and 10^4^ at 40 °C, indicating a weak reverse transcriptase capability of Bst-like Gss-polymerase. The efficacy of reverse transcription at 60 °C was much lower, possibly due to the Afl RT thermal inactivation. Nevertheless, despite reduced activity, we succeeded in detecting 104 copies of MS2 genomic RNA. Accordingly, the temperature range of 40–50 °C proved to be the most effective for reverse transcription in RT-qLAMP.

#### 3.3.3. Processivity of Afl RT

**Figure 9:**
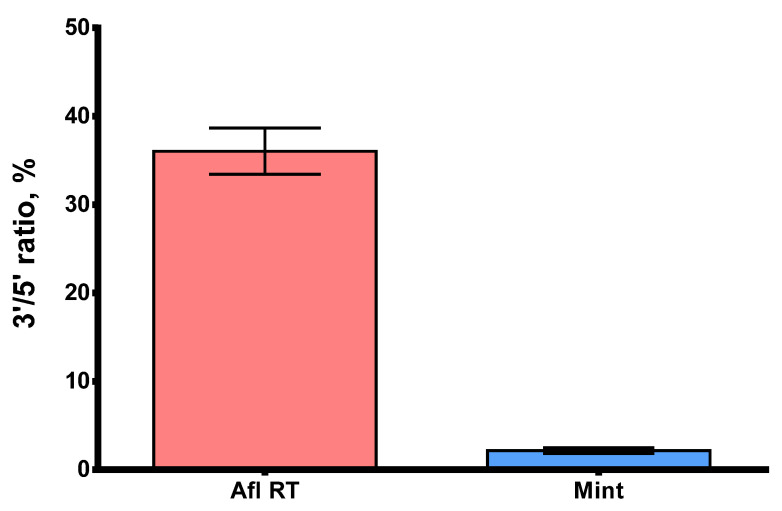
The 5′/3′-ratio of cDNA synthesized by Afl RT and M-MuLV RT. MS2 genomic RNA (3569 nt) served as a template for reverse transcription. Each reaction was run in three technical repeats. The 5′/3′-ratio of cDNA ends was determined using ddPCR with primers for 5′- and 3′-ends of MS2 genomic RNA.

Processivity is a significant parameter that defines the ability of reverse transcriptases to synthesize full-length cDNA fragments. The presence of short, partially elongated cDNA sequences may pose challenges in further analysis, particularly in NGS. Within this context, GC-rich and highly structured RNA molecules are of special interest, with MS2 genomic RNA being a suitable model for studying such complex templates. Here, we studied the processivity of Afl RT using MS2 genomic RNA (3569 nt) as a template for long cDNA synthesis. The length of cDNA products was assessed using droplet digital PCR with two sets of primers and TaqMan probes for both 5′- and 3′-ends of the cDNA (Table 1). The 5′/3′-ends ratio served as a surrogate marker of Afl RT processivity: a processive enzyme synthesizing longer cDNA molecules should have a 5′/3′-ends ratio closer to 100%. The results of the processivity assay are presented in Figure 9.

The 5′/3′-ratio of cDNA ends for Afl RT was close to 36.0%, indicating that 1/3 of cDNA molecules were full-length, which was significantly higher than 2.2% for M-MuLV RT. Consequently, the processivity of Afl RT surpassed that of M-MuLV RT by several folds and resembled that of other GII RTs.

## 4. Discussion

Reverse transcriptases were first discovered in retroviruses, with retroviral RTs becoming model enzymes for investigating the conversion of RNA to cDNA. It is worth noting that retroviral RTs have also become crucial instruments for the study of RNA, where the conversion of RNA into more stable and convenient cDNA is required. Monomeric M-MuLV RT and its mutants, known for their heightened thermal stability, processivity, and inhibitor tolerance, are the most commonly used retroviral RT enzymes in commercial applications. Although dimeric AMV RTs and HIV-1 RTs are more thermostable than wild-type M-MuLV RTs, all of these enzymes are inactivated when incubated for several minutes at temperatures higher than 70–75 °C [34,35,36]. The processivity and fidelity of these retroviral RTs are relatively low, being in the range of 14–97 b and one mismatch per 10^4^ incorporated nucleotides, respectively [37,38]. These parameters limit the usage of conventional RTs in practical applications where uniform and precise cDNA synthesis is crucial, for example, in RNA-seq, especially in a single-cell format. The short length of cDNA molecules leads to the under-representation of reads corresponding to the 5′-ends of mRNA molecules. Mismatches and other altercations in reads, such as indels, increase noise and decrease the sensitivity in detecting rare events. For these reasons, there is a high demand for novel, more thermostable, and accurate RTs.

Compared to RTs from retroviruses that have been extensively studied and introduced in biotechnology, reverse transcriptases from prokaryotes remain a dark matter and require further investigation. A recent phylogenetic analysis divided prokaryotic RTs into 42 classes, with only 11 classes having their functions uncovered [22]. For instance, retrons discovered 30 years ago were not found to be involved in anti-phage defense until 2020 [7]. This lack of knowledge is striking compared to DNA polymerases of other families involved in replication and DNA repair. Thus, there is a need for systematic biochemical studies to elucidate the in vivo role of these enzymes.

Among bacterial RTs, the most abundant are enzymes from type II introns, or GII RTs, accounting for approximately 65% of all putative RTs [22]. Several GII RTs were simultaneously cloned, purified, and biochemically characterized, yielding two exceptionally processive, precise, and relatively thermostable RTs from *T. elongates* (TGIRT) and *E. rectale* (Marathon RT) [14,15]. However, these results were insufficient to systematically compare RTs from type II introns with retroviral counterparts. Moreover, some properties of GII RTs remain undescribed, including the optimal reaction buffer, which is crucial to achieve the best performance in practical applications.

Given that this study was aimed at finding novel enzymes that could have potential for biotechnology applications, we focused on GII RTs from thermophilic bacteria. For that reason, we excluded RTs with any additional domains and considered only enzymes with a classical domain structure with fingers, palm, and an X-domain (thumb) [11]. GII RT from a thermophilic bacterium, *Anoxybacillus flavithermus*, was found to meet all the above criteria and was selected for further biochemical analysis.

GII RTs are notoriously known for their insolubility, purification issues, and low yield after isolation [14,15,16,39]. In bacteria, the enzymes are present as ribonucleoproteins, with the protein component tightly binding the respective coding RNA. Additionally, GII RTs have a high positive charge and an isoelectric point close to 9–10. These factors are believed to result in a tendency to form insoluble conglomerates upon heterologous expression in *E. coli* cells. Initial attempts to purify GII RT enzymes resulted in a significant loss of specific activity and high contamination with E. coli proteins [16,39]. Expression at 18–20 °C, often yielding proteins in a soluble fraction, was also unsuccessful. Later, the insolubility problem was partially solved by fusing GII RTs either with a maltose-binding MalE protein via a rigid alanine linker in TGIRT [14] or with a SUMO tag in Marathon RT [15]. Interestingly, RTs fused with MalE via a common flexible linker remained insoluble. In this work, we succeeded in purifying Alf RT without any solubility tags, using only a 2 M urea as a mild chaotropic agent. A similar approach was used by Haack et al. to purify GII RT from *T. elongates* [40]. The difference was that the authors used 2 M urea and MBP tagging simultaneously. It is possible that a slight disruption of interactions with RNA molecules, proteins, and other cell components was enough to purify GII RT without fusion with additional domains for increasing solubility. However, with GII RTs having similar pI, net charge, and domain structure, this discrepancy between our results and the observations of other research groups should be specifically clarified. 

The previous works devoted to the characterization of GII RT were mainly aimed at demonstrating the superior thermostability, processivity, and template-switching activity of these enzymes. A proper description of these enzymatic properties is essential for practical applications. However, of practical importance is also the optimization of the reaction buffer. Suboptimal conditions can decrease a reaction yield and the length of cDNA products. This work is the first attempt to examine the relative activity of a GII RT in the presence of various salts and divalent cations. An interesting observation is that Afl RT displayed more activity in the presence of Mn^2+^ than conventional Mg^2+^. According to our knowledge, such a result has not been reported before for any other GII RT. A similar effect was described before for the retroviral M-MuLV RT [41]. The fidelity of DNA polymerases and RTs is generally lower in the presence of uncanonical cofactors [31,42]. Thus, Mg^2+^ ions are preferable for correct synthesis, especially given the possible application of GII RTs in single-cell RNA-seq. 

Similar to other GII RTs characterized, Afl RT has demonstrated a high degree of processivity. It fully elongated 1/3 of all cDNA molecules using MS2 genomic RNA as a template. This processive synthesis, being necessary for the propagation of group II introns in cells, proves to be a characteristic feature of all GII RTs. The same observation can be made regarding thermostability: enzymes derived from moderate thermophiles living at 50–60 °C are relatively thermostable in vitro, as well. Although the optimal reaction temperature for Afl RT was lower than that for TGIRT and Marathon RT, RT-LAMP with Afl RT demonstrated good sensitivity in the optimal buffer for Gss-polymerase. Thus, Afl RT may become an alternative to common commercial retroviral RTs.

Mention should be made of several limitations of the present study. It would be beneficial to directly compare the new Afl RT with TGIRT and Marathon RT to gain more insight into the relationship between the structure and enzymatic properties of GII RTs. GII RTs are known for their strong terminal transferase activity, a property that has not been tested in Afl RT. Moreover, the suitability of Afl RT for RT-PCR should be evaluated, and we plan to conduct the necessary experiments in the future.

To summarize, we have cloned a new reverse transcriptase from a group II intron of a thermophilic bacterium, *Anoxybacillus flavithermus*. The biochemical characterization and testing in reverse transcription and RT-LAMP have revealed the significant biotechnological potential of the novel enzyme, Afl RT.

## 5. Conclusions

This work reports on biochemical characterization and practical application testing of a novel RT derived from a thermophilic bacterium called *Anoxybacillus flavithermus*. The cloned Afl RT exhibited a higher degree of thermostability compared to retroviral RTs and demonstrated enhanced processivity. Moreover, this enzyme also allowed us to detect up to 10 copies of an RNA template in reverse transcription and RT-LAMP. Consequently, Afl RT holds great promise for various biotechnological applications.

## Figures and Tables

**Table 1:** List of oligonucleotide primers and probes.

Name	5′-Sequence-3′
MS2-FAM	FAM-GCCATTTTTAATGTCTTTAGCGAG
MS2-2-F3	TGCCTGTAAGGAGCCTGAT
MS2-2-B3	TGAGCGGATACGATCGAGAT
MS2-2-LB	GTCTATACCAACGGATTTGAGCC
MS2-2-LF	GCATCCGATTCCATCTCCGAT
MS2-2-FIP	GCCAGACGCTGGTTGATCGATTAAGGGGTCGGTGCTTTCA
MS2-2-BIP	GGTTCGCTTGCGACGATAGACTTCTGGTGGGAGAAAACTCCA
MS2-5-F2	GCCATTTTTAATGTCTTTAGCGAG
MS2-5-R2	AGGAATGGAATTCCGGCT
MS2-5-P	FAM-TCCCTCGACGCACGCTCCTGCT-BHQ1
MS2-3-F	TGTGCTCGAAAGAGCACG
MS2-3-R	GAATCCCGGGTCCTCTCT
MS2-3-P	HEX-AGCGGTCCGGCTCCA-BHQ1

**Table 2:** Properties of several RTs from retroviruses and group II introns.

RT	GenBank ID	Length, a.a.	MW, kDa	pI	Net Charge at pH 7.0
M-MuLV RT	NP_955591.1	671	74.6	8.67	7.73
HIV-1 RT	1LWF_A	560	64.5	8.69	5.53
TeI-4c RT	BAC08074.1	564	64.7	10.03	45.3
Gs1-2c	AAT72329.1	420	48.6	10.59	36.21
Afl RT	WP_012575961.1	418	49.7	10.66	42.41

## Data Availability

The data presented in this study are available on request from the corresponding author.

## Supplementary Materials

The following supporting information can be downloaded at: https://www.mdpi.com/article/10.3390/biom14010049/s1, Figure S1. Expression and purification of Afl RT. The enzyme was expressed in *E. coli* strain BL-21 (DE3) pLysS and purified using affinity and ion-exchange chromatography. Lane assignment: M—Precision Plus Protein standards (Bio-Rad, Hercules, CA, USA), 1—*E. coli* lysate before expression of Afl RT, 2—crude lysate after expression, 3—soluble protein fraction after lysis, 4—resuspended proteins after (NH4)2SO4 precipitation, 5—Afl RT after affinity chromatography, 6—Afl RT after anion-exchange chromatography, 7—purified Afl RT after dialysis. Afl RT is marked with an arrow. Reference [43] are cited in the supplementary materials.
